# Exploratory factor analysis and Rasch analysis to assess the structural validity of the Adult Social Care Outcomes Toolkit Proxy version (ASCOT-Proxy) completed by care home staff

**DOI:** 10.1007/s11136-024-03631-1

**Published:** 2024-03-20

**Authors:** Stacey Rand, Ann-Marie Towers, Stephen Allan, Lucy Webster, Sinead Palmer, Rachael Carroll, Adam Gordon, Gizdem Akdur, Claire Goodman

**Affiliations:** 1 Personal Social Services Research Unit (PSSRU), University of Kent, Canterbury, UK; 2 Centre for Health Services Studies (CHSS), University of Kent, Canterbury, UK; 3 University of Nottingham, Nottingham, UK; 4 University of Hertfordshire, Hatfield, UK

**Keywords:** Older adults, Care homes, Quality of life, Outcomes, Social care

## Abstract

**Purpose:**

Rasch analysis and exploratory factor analysis (EFA) were used to evaluate the structural validity of the ASCOT-Proxy measures completed by staff on behalf of older adults resident in care homes, by comparison to the ASCOT-SCT4, the measure of social care-related quality of life (SCRQoL) from which the ASCOT-Proxy was developed.

**Methods:**

EFA was conducted on the ASCOT-SCT4 and the two ASCOT-Proxy measures (Proxy-Proxy, Proxy-Resident), to determine if they retained the single factor of the original ASCOT-SCT4 measure found in samples of older community-dwelling adults. Rasch analysis was also applied to measures with a single factor structure in the EFA.

**Results:**

ASCOT-Proxy-Resident had a single factor structure, as did the original ASCOT-SCT4 (also, found in this analysis when completed by care home staff). The ASCOT-Proxy-Proxy had a two factor structure. Rasch analysis of ASCOT-Proxy-Resident and ASCOT-SCT4 had an acceptable model fit, internal consistency and met the assumptions of unidimensionality and local independence. There was evidence of less than optimal distinguishability at some thresholds between responses, and low frequency of rating of the ‘high level needs’.

**Conclusion:**

The ASCOT-Proxy-Resident is a valid instrument of SCRQoL for older adults resident in care homes, completed by staff proxies. Due to the two-factor structure, which differs from the original ASCOT-SCT4, we do not recommend the use of the ASCOT-Proxy-Proxy measure, although collecting data as part of the ASCOT-Proxy questionnaire may support its feasibility and acceptability. Further qualitative study of how care home staff complete and perceive the ASCOT-Proxy is encouraged for future studies.

**Supplementary Information:**

The online version contains supplementary material available at 10.1007/s11136-024-03631-1.

## Introduction

Routine standardised data collections from older adults resident in care homes are conducted in some countries: for example, data are collected using Minimum Data Set (MDS) 3.0 in the US [1, 2] and the International Resident Assessment Instrument (InterRAI) in various countries [3]. These resident-level data collections, known as minimum data sets (MDS), are used for a range of purposes, from direct care to analysis that informs policy, planning, funding and delivery of services. In the UK, however, there is currently no systematic routine collection of data in a centralised, aggregated form. Instead, there are various separate health and social care data collections held in different formats, by different agencies. A current UK policy aim is to move towards greater standardisation, wider adoption of digital data collection, and linkage of individual-level data to maximise use [4].

In this context, the Developing resources And minimum data set for Care Homes' Adoption (DACHA) study is a programme of research to develop and test the feasibility of a resident-level UK MDS [5, 6]. One DACHA project work package was an individual-level pilot data collection from older adult care homes in England, via digital care records and linked data [5]. This drew on data collected in routine inpractice by participating homes, as well as from additional measures added by software providers, and completed by care home staff. These included resident-level quality of life (QoL), since QoL was a priority for inclusion in the MDS to reflect residents’ and families’ priorities [6] and to address the critique that existing MDS focus too narrowly on clinical, health and functioning data [2, 6]. Following consultation with stakeholders [7], five QoL measures were selected: EQ-5D-5L Proxy 2 [8], QUALIDEM [9, 10], ICECAP-O [11, 12], QoL item (5-point scale), and the two QoL measures from the Adult Social Care Outcomes Toolkit for proxy completion (ASCOT-Proxy) [13]. This paper focuses on the two ASCOT-Proxy measures and, specifically, their structural validity,^1^ since the measures have been relatively recently developed and DACHA was the first data collection from care staff on behalf of residents. The psychometric properties of the other individual-level QoL measures in DACHA, as well as construct validity by hypothesis testing of the ASCOT-Proxy measures, are reported elsewhere.

ASCOT-Proxy is a questionnaire that collects data for two separate measures of social care-related QoL (SCRQoL) (see www.pssru.ac.uk). It was developed and adapted from ASCOT-SCT4, a self-report measure of SCRQoL that was originally designed for older adults living at home [15]. ASCOT-SCT4 has been found to be valid and reliable for adults of all ages, with a range of support needs [16], translated into various languages [17–19], and adapted for mixed-methods data collection in care homes (CH4) [20, 21]. In care homes, data collection using ASCOT-SCT4 is often not feasible. An estimated 70% of UK care home residents have dementia [22] and many are unable to self-report QoL, even with flexible methods. The CH4 (another adapted version of ASCOT-SCT4) may also not always be feasible since its data collection method is resource-intensive [20]. This is why the ASCOT-Proxy was developed, with family carers and care workers, to enable data collection for people unable to self-report [13, 23]. Proxy respondents are asked to rate the ASCOT-Proxy items from the *proxy-person perspective* (i.e. what the proxy thinks the person thinks) and *proxy-proxy perspective* (i.e. what the proxy thinks about the person’s QoL). These ratings generate the two measures of proxy-report SCRQoL: ASCOT-Proxy-Resident and ASCOT-Proxy-Proxy.

The DACHA study was the first data collection of ASCOT-Proxy from care home residents. The study used staff proxy report, since a previous study found that data collection from family proxies with the CH4 led to high levels of missing data, whereas staff report gave a more complete data [20]. Previous studies of proxy report of QoL provide evidence of differences, albeit small, in rating by proxy ‘type’ (e.g., staff vs. family) [24], so a consistent approach to proxy report by direct care staff, who knew the resident well, was adopted. Since ASCOT-proxy was developed [13], there has been one study of its psychometric properties, in a sample of family carers of community-dwelling people with dementia [25]. This study found that one measure, ASCOT-Proxy-Person (Resident), had acceptable properties; however, the other measure, ASCOT-Proxy-Proxy, did not fit to the expected unidimensional scale based on the single factor structure of the original ASCOT-SCT4 [15]; instead, it was found to have a two-factor structure [25]. There were also issues with the rating scale for two QoL domains (*Food and drink* and *Personal comfort and cleanliness*), which warranted further investigation [25]. Due to these findings, especially since they were based on family carer proxy-report for people with dementia living at home, there is a need for further evaluation of the measure, in general, and also for proxy report by care home staff, as in the DACHA study [5].

Therefore, the aim of this analysis was to evaluate the structural validity of the ASCOT-Proxy measures, as collected in the DACHA study by care home staff, using exploratory factor analysis and Rasch analysis. This forms part of the process of deciding whether and how to recommend the measure’s inclusion in a MDS, alongside evaluation of other psychometric properties (e.g., construct validity by hypothesis testing) and acceptability to care staff, which are reported elsewhere. Evaluation of ASCOT-Proxy’s structural validity, as well as other psychometric properties, is also important for guiding its future development and use in routine data collection, evaluation and research. This paper will inform the understanding of the collection of SCRQoL by care home staff proxies using ASCOT-Proxy, with comparison to the ASCOT-SCT4, also collected from care home staff proxies.

## Methods

### Data collection

#### Study 1 (ASCS, ASCOT-SCT4)

ASCOT-SCT4 [15], the original ASCOT measure from which ASCOT-Proxy was developed, is routinely collected in the English Adult Social Care Survey (ASCS) [26]. The ASCOT-SCT includes nine items, covering eight domains: Control over daily life, Social participation and involvement, Occupation (doing things I value and enjoy), Personal comfort and cleanliness, Food and drink, Accommodation comfort and cleanliness, Personal safety and Dignity. For each item, there are four statements that relate to the person’s ideal state (all needs and preferences met), no needs, some unmet needs, and high-level unmet needs (risk to health).

The ASCS is an annual survey of adults, aged 18 or over, across England, who receive local authority funded or managed services. The majority of responses are collected by postal survey using a self-complete questionnaire (see [26] for detail). Although the ASCS guidance states anyone who lacks capacity ought to be excluded from the sample [26], approximately 9% of ASCS data is completed by proxy. The majority of proxy responses are completed by family and friends but around 1 in 15 are completed by care staff. ASCS datasets only collect data on whether it is completed by proxy, and whether that proxy is care staff or family/friend. No further information is collected on proxies and the guidance does not specify who ought to act as a proxy.

For the analysis presented here, we extracted ASCOT-SCT4 care home staff proxy responses from the 2011 to 2022 ASCS for older adults, aged 65 or over, who were resident in a care home. This yielded a sample of 697.^2^ We consider these data as a comparator, as there has not previously been a formal assessment of structural validity of the ASCOT-SCT4 collected by care home staff proxy response and the DACHA study did not include the –SCT4, to avoid duplication and minimise care home staff burden.

#### Study 2 (DACHA, ASCOT-Proxy)

**Fig. 1:**
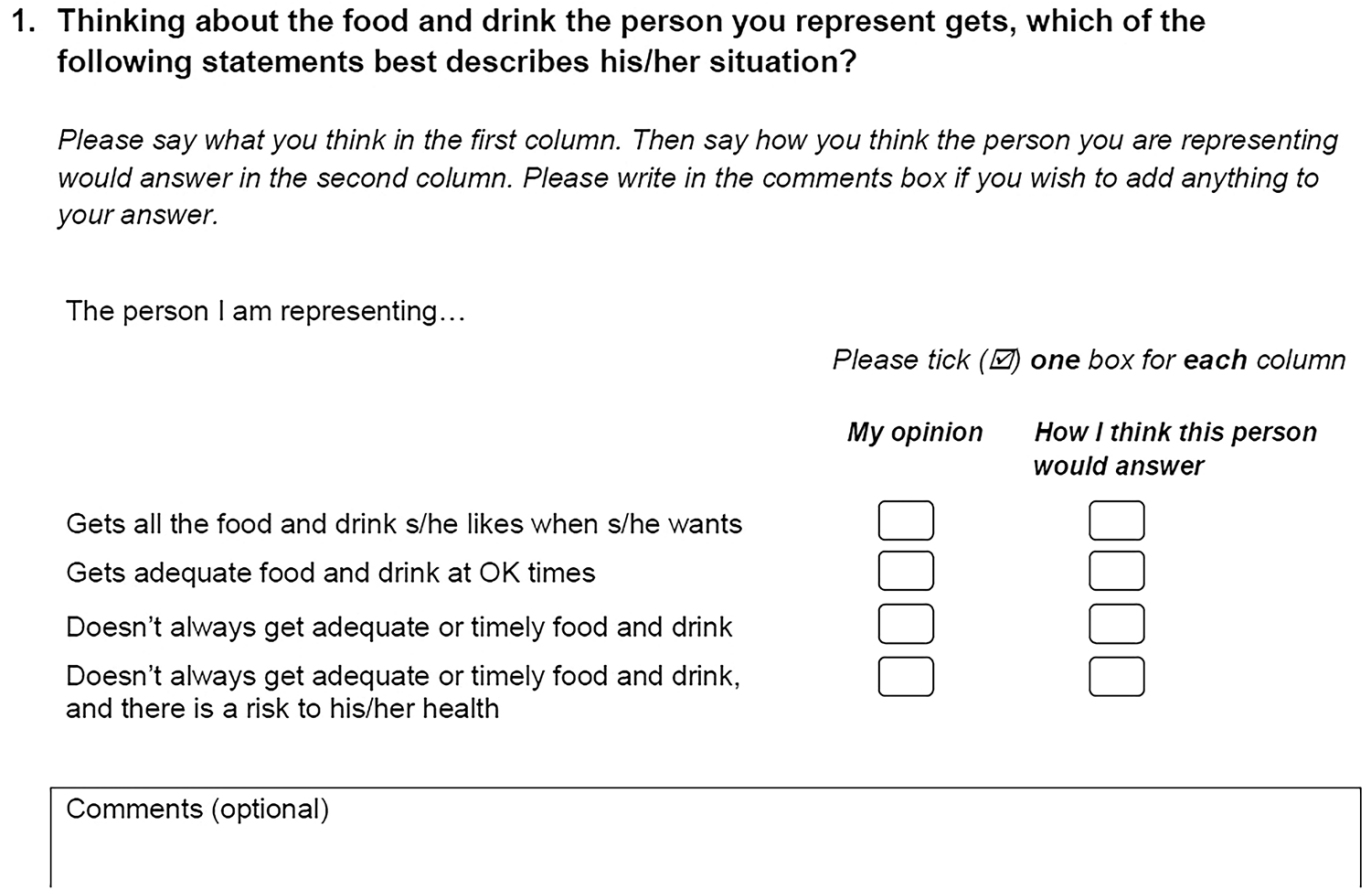
Example ASCOT-Proxy item (food and drink). © PSSRU at the University of Kent. www.pssru.ac.uk/ascot

ASCOT-Proxy, along with other QoL measures included in the DACHA study and other measures (see study protocol for full details [5]), was added into digital care record software by two care software providers, working with the DACHA study. ASCOT-Proxy covers the same domains as ASCOT-SCT4, and uses the same four item response options; however, the question wording is adapted to indicate the question is being asked of a proxy and allows for two ratings for each domain for the ASCOT-Proxy-Resident and ASCOT-Proxy-Proxy [13] (Fig. 1).

Care homes were eligible to participate if they were using one of the two software systems, and in one of these three integrated care systems (ICSs) in England, which were selected to represent a range of geographic/regional, socio-economic and organisational contexts. In the participating care homes, all permanent residents, aged 65 years or older, were eligible to take part. We excluded residents thought by staff to be in their last weeks of life. The study included residents with and without the capacity to consent to the study. Where residents were not able to consent for themselves, a nominated or personal consultee was consulted to advise on the views and feelings they believed the resident would have. ASCOT-Proxy was completed by a staff member for all participating residents, with the staff member being someone who knew the resident well, with or without consultation with the resident based on the staff member’s judgement.

Data were entered by care home staff between March and May 2023, and extracted by software provider 1 on 27 June 2023, and software provider 2 in three batches, on 14 June, 17 August, and 28 September 2023. The data considered in this analysis excludes four participating care homes from software provider 2, due to an issue with flagging data entry that affected this data extract. In the analysis presented here, we only considered data where all ASCOT-Proxy-Resident (*n* = 462) and ASCOT-Proxy-Proxy (*n* = 511) items were completed.

### Statistical analysis

Exploratory factor analysis (EFA) followed by Rasch analysis were applied to ASCOT-Proxy (Study 2) to determine the structural validity of the ASCOT-Proxy completed by care home staff, with comparison to ASCOT-SCT4, the original ASCOT measure from which the ASCOT-Proxy was developed, completed also by care home staff (Study 1).

Analyses were conducted on complete cases (i.e., no imputation of missing values) in STATA 16, except for Rasch analysis in WINSTEPS 3.91.1 [27].

#### Exploratory factor analysis

The Kaiser–Meyer–Olkin (KMO) measure of sampling adequacy [28] and Bartlett’s test of sphericity [29] were used to indicate appropriateness of factor analysis for each measure. Ordinal exploratory factor analysis (EFA) was conducted because ASCOT-SCT4 and ASCOT-Proxy item ratings are ordinal. The user-written command *polychoric* was used to calculate polychoric correlations [30, 31] and EFA was run on the polychoric correlation matrices. To guide retention of factors in the EFA, Horn’s parallel analysis with the user written command, *paran*, which uses principal component analysis (PCA), without rotation, was applied [32–36]. Randomly generated eigenvalues were estimated in 5,000 random correlation matrixes, using the 95th percentile, and factors were retained where the observed exceeded random principal component Eigenvalues [33, 37].

#### Rasch analysis

Rasch analysis compares observed (typically, ordinal) data against a unidimensional mathematical measurement model (‘Rasch model’). This model assumes that the probability of affirming an item is a logistic function of the difficulty of the item (*item*) and the ability of the person (*person*). By fitting QoL data to the Rasch model, ordinal data is converted into interval level measurement with log odds as the unit interval [38]. Rasch analysis can be applied to guide item reduction [38] or, as in the case of this analysis, for the development and testing of existing or adapted measures [39, 40]. Because ASCOT-SCT4 and ASCOT-Proxy items have ordered response options that are unique to each item, we fitted data using a partial credit model [41]. Adjacent response categories were collapsed when there was evidence of disordered thresholds using visual inspection of ordered category probability curves [42], with the Rasch model re-calculated on data with collapsed categories.

The Rasch model assumes local independence and unidimensionality, i.e., the measure relates to a single latent dimension or construct [38]. To evaluate the fit of observed data to the Rasch model (with collapsed categories, as above), we used principal component analysis of standardised residuals to assess whether residual variance is random after considering the primary measurement dimension, as would be expected if the data fitted to a single latent dimension. The criteria applied was that first principal component of the residuals should have an Eigenvalue of less than 2.0 [42, 43]. To evaluate local independence, we examined standardised residual correlations for items; correlations < 0.20 were taken to indicate local independence of items [44]. Finally, an item separation index of > 1.5 was taken to indicate adequate internal consistency [42] and that the sample can be divided into two strata by the measure, i.e., low vs high QoL [45].

To evaluate how well observed data fitted to the Rasch model, a number of overall fit summary statistics were considered. A non-significant overall summary Chi-square interaction fit statistic was taken to indicate good fit to the Rasch model. The standard deviation of the item summary residual statistic was evaluated against the criterion of one indicating ‘perfect fit’, with values of < 1.5 deemed to be acceptable [46]. Since the Chi-square test is sensitive to larger samples, as in the two studies we report here, the summary residual statistics were preferred when the Chi-square results differed [44]. To further understand any misfit to the Rasch model, INFIT and OUTFIT mean square statistics for each item were considered, with values in the range of 0.5 to 1.5 indicative of good fit.

Functionality of the ASCOT rating scale of four ordered categories was assessed using the following criteria: (1) ≥ 10 cases per response category; (2) average measures and category thresholds increase by response category, with step difficulties increasing between 1.4 to 5.0 logits per category; and (3) OUTFIT MNSQ of < 2.0 for each response category [47]. If the first criteria did not hold (i.e., *n* < 10 per response category) and the other criteria were also not met, the finding was taken to be inconclusive due to small numbers, by response.

## Results

The sample descriptive statistics are shown in Table 1.

**Table 1:** Sample characteristics

	Study 1 ASCS	Study 2 DACHA
	Frequency, *N* (%)	Frequency, *N* (%)
*Sex*		
Female	450 (64.6%)	155 (29.6%)
Male	247 (35.4%)	67 (12.8%)
Missing	0 (0%)	301 (57.6%)

	Mean, Std. Dev (min. to max.)	Mean, Std. Dev (min. to max.)
ADLs with difficulty^a^	5.74, 2.44 (0 to 8)	n/a
Barthel Index	n/a	8.47, 6.01 (0 to 20)
ASCOT-SCT4	19.90, 3.12 (8 to 24)	n/a
ASCOT-Proxy-Proxy	n/a	19.23, 4.08 (0 to 24)
ASCOT-Proxy-Resident	n/a	19.88, 3.35 (9 to 24)

^a^A sum of the number of eight activities of daily living (ADLs), where the resident has difficulty and/or requires help: indoor mobility, getting in/out of bed or a chair, eating, dealing with paperwork or finances, bathing, getting un/dressed, using the toilet, washing hands and face

### Exploratory factor analysis

The Kaiser–Meyer–Olkin measure of sampling adequacy indicated that the correlations between items were sufficient for factor analysis (KMO = 0.81 for ASCOT-SCT4 and KMO = 0.85, 0.81 for ASCOT-Proxy-Resident and -Proxy, respectively). Likewise, Barlett’s test of sphericity indicated a rejection of the null hypothesis that items were not inter-correlated for all three measures (ASCOT-SCT, χ^2^ (28) = 820.52, *p* < 0.001; ASCOT-Proxy-Resident χ^2^ (28) = 1289.30, *p* < 0.001; ASCOT-Proxy-Proxy, χ^2^ (28) = 964.43, *p* < 0.001).

Horn’s parallel analysis indicated a single factor solution for the ASCOT-SCT4 and ASCOT-Proxy-Resident (i.e., the observed Eigenvalue exceeded the random principal component Eigenvalue only for the first factor), but a two factor solution for ASCOT-Proxy-Proxy. For ASCOT-SCT4 (Table 2) and ASCOT-Proxy-Resident (Table 3), all items loaded onto a single factor with factor loadings ≥ 0.40 [48]. There was high unique variance (≥ 0.60) for four ASCOT-SCT4 domains (Accommodation, Personal safety, Social and Dignity), and two domains had high unique variance in the ASCOT-Proxy-Resident EFA (Personal safety, Dignity).

**Table 2:** Exploratory factor analysis for ASCOT-SCT4 (Study 1)

	Factor loading	Uniqueness
1. Food & drink	0.66	0.57
2. Home comfort & clean	0.61	**0.63**
3. Personal comfort & clean	0.67	0.56
4. Social participation	0.61	**0.63**
5. Occupation	0.70	0.51
6. Control over daily life	0.66	0.57
7. Personal safety	0.59	**0.65**
8. Dignity	0.49	**0.76**

Items with uniqueness ≥ 0.60 shown in bold

**Table 3:** Exploratory factor analysis for ASCOT-Proxy-Proxy and –Resident (Study 2)

	ASCOT-Proxy-Proxy				ASCOT-Proxy-Resident	
	Factor One		Factor Two			
	Rotated loading	Uniqueness	Rotated loading	Uniqueness	Un-rotated loading	Uniqueness
1. Food & drink			0.627	**0.610**	0.696	0.515
2. Home comfort & clean			0.705	0.392	0.853	0.272
3. Personal comfort & clean			0.704	0.421	0.763	0.418
4. Social participation	0.747	0.456			0.744	0.447
5. Occupation	0.856	0.223			0.790	0.377
6. Control over daily life	0.712	0.447			0.734	0.464
7. Personal safety			0.674	0.506	0.607	**0.631**
8. Dignity	0.413	**0.708**			0.591	**0.650**

Items with uniqueness ≥ 0.60 shown in bold

For the ASCOT-Proxy-Proxy, a two-factor solution with promax rotation is also reported in Table 3. All items loaded with factor loadings ≥ 0.40. The first factor comprised the higher order domains of quality of life that relate to QoL needs beyond basic care (i.e., Control, Social, Occupation), as well as Dignity or whether the process care delivery ensures the person’s sense of sense and dignity is maintained. The second factor included the four basic domains of care-related QoL, which relate to the basic actions of care to support individual daily activities and needs (i.e., supporting food and drink, personal comfort and cleanliness, home comfort and cleanliness, and personal safety).

### Rasch analysis

A single factor structure was expected based on previous studies of the ASCOT-SCT4 from which the ASCOT-Proxy was developed, which has a single factor structure [15], and was also supported by the analysis presented here with ASCOT-SCT4 data collected by care home staff proxy report (Study 1). However, only the EFA for ASCOT-Proxy-Resident supported a single factor structure. Therefore, we proceeded to Rasch analysis with ASCOT-Proxy-Resident (Study 2) only, alongside ASCOT-SCT4 (Study 1) for comparison.

**Fig. 2:**
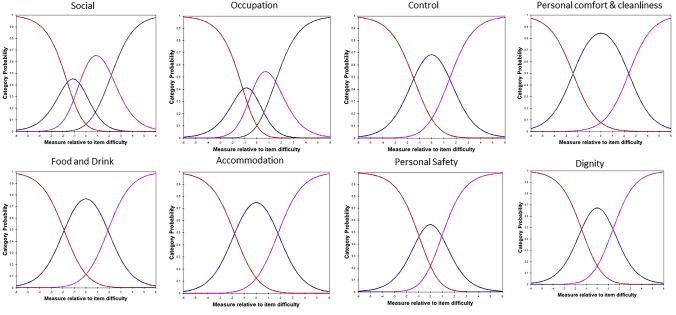
Revised category probability curves for ASCOT-SCT4 (Study 1)

**Fig. 3:**
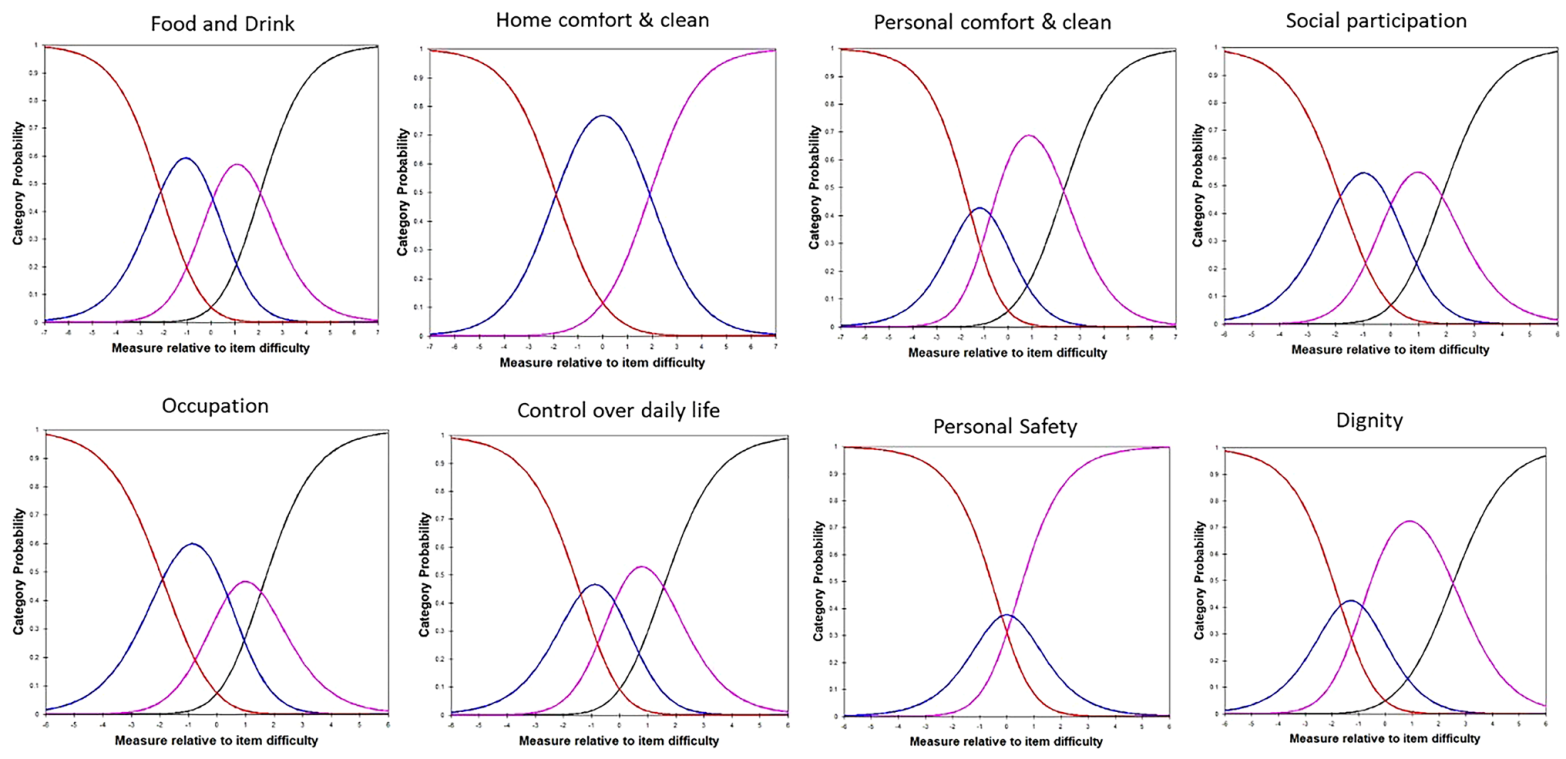
Revised category probability curves for ASCOT-Proxy-Resident (Study 2)

For the ASCOT-SCT4, the category probability curves indicated disordered thresholds for six of eight domains (all except for *Social* and *Occupation*), with the probability curve for some needs “buried” under the other curves. Therefore, high-level and some needs responses were collapsed for these six domains. Visual inspection of the category probability curves indicated that no further evidence of disordered thresholds (Fig. 2). For ASCOT-Proxy-Resident, the category probability curves indicated disordered thresholds for two of the eight domains. The probability curve for some needs (*Home comfort and cleanliness, Personal safety*) were “buried” under the other curves, so high-level and some needs were combined for these two items. The revised category probability curves are shown in Fig. 3, with no further evidence of disordered thresholds.

The first principal component of the residuals’ Eigenvalue was less than the criterion of 2.0 for both ASCOT-SCT4 (1.56) and ASCOT-Proxy-Resident (1.85), which indicated good fit of observed data to the unidimensional Rasch model. Local independence of items (i.e., standardised residual correlations of < 0.20) was confirmed for the ASCOT-SCT4. There was one positive correlation > 0.20 between *Home* and *Personal comfort and cleanliness* for ASCOT-Proxy-Resident (0.28), although the other correlations between item residuals were negative (i.e., no evidence of local dependence). The item separation index was above 1.5 for both measures (Table 4), which indicates adequate internal consistency.

**Table 4:** Model fit statistics for ASCOT-SCT4 and ASCOT-Proxy–Resident

	Study	Overall model fit	Item fit resid. Mean (Std. Dev.)	Person fit resid. Mean (Std. Dev.)	Item separation index
ASCOT-SCT4	1	χ^2^ (6,179 df) = 6061.74, *p* = 0.854	0.00 (1.28)	2.43 (1.72)	13.62
ASCOT-Proxy-Resident	2	χ^2^ (3,131 df) = 4575.43, *p* < 0.001	0.00 (0.98)	2.46 (1.82)	9.68

The overall Chi-square statistic indicated a good overall fit to the Rasch model for ASCOT-SCT4 (*p* = 0.850), but not ASCOT-Proxy-Resident (*p* < 0.001). This fit statistic can be sensitive to large sample sizes, like the two samples of study. Therefore, we also considered the summary residual statistics for items and persons, with preference for these statistics where results differ [44]. For both ASCOT-SCT4 and ASCOT–Proxy-Resident, the standard deviation of the item summary residual statistic was less than the criterion of 1.5, which indicates good fit. Evaluation of individual item statistics also indicated good fit for all items across both ASCOT-SCT4 and ASCOT–Proxy-Resident, with INFIT and OUTFIT mean square values within the acceptable criteria range of 0.50 to 1.50 (Table 5).

**Table 5:** Item statistics

	Item difficulty	SE	INFIT MNSQ	OUTFIT MNSQ	Point-measure correlation
*ASCOT-SCT4*					
1. Food and drink	−0.79	0.10	1.02	1.12	0.51
2. Home comfort & clean	−1.22	0.10	1.01	1.01	0.48
3. Personal comfort & clean	−0.54	0.09	0.94	0.92	0.60
4. Social participation	0.48	0.07	1.04	1.02	0.65
5. Occupation	1.12	0.06	0.84	0.84	0.74
6. Control over daily life	2.58	0.08	0.93	0.94	0.74
7. Personal safety	−1.57	0.13	0.96	1.14	0.39
8. Dignity	−0.07	0.09	1.21	1.26	0.47
*ASCOT-Proxy-Resident*					
1. Food and drink	−0.71	0.10	1.08	1.30	0.59
2. Home comfort & clean	−0.60	0.11	0.83	0.76	0.65
3. Personal comfort & clean	−1.55	0.12	0.95	0.87	0.57
4. Social participation	0.40	0.08	0.96	0.94	0.72
5. Occupation	1.32	0.07	0.79	0.79	0.80
6. Control over daily life	1.49	0.07	0.95	0.95	0.77
7. Personal safety	−0.53	0.12	1.09	1.30	0.46
8. Dignity	0.17	0.09	1.26	1.30	0.61

The rating scale diagnostics against Linacre’s criteria [47] are shown in Table 6 and 7. First, even after combining categories due to evidence of disordered thresholds based on visual inspection of category probability curves, four ASCOT-SCT4 and three ASCOT-Proxy-Resident items had < 10 endorsements for ‘high-level needs’ and/or ‘some needs’.

**Table 6:** Rating scale diagnostics for ASCOT-SCT4

	Observed count	Observed average	OUTFIT MNSQ	Rasch-Andrich threshold	Category measure
*1. Food and drink*					
High-level or some needs^a^	9	−0.75	0.81	NONE	−3.79
No needs	188	1.23	1.22	−1.88	−0.79
Ideal state	481	2.49	0.99	1.88	2.20
*2. Home comfort & clean*					
High-level or some needs^a^	6	−0.47	1.00	NONE	−4.12
No needs	144	0.98	1.01	−1.78	−1.22
Ideal state	531	2.42	0.99	1.78	1.68
*3. Personal comfort & clean*					
High-level or some needs^a^	8	−1.02	0.74	NONE	−4.03
No needs	267	1.34	0.90	−2.39	−0.54
Ideal state	397	2.72	0.99	2.39	2.95
*4. Social participation*					
High-level needs	19	−0.39	1.05	NONE	−2.43
Some needs	70	0.69	0.90	−1.60	−0.64
No needs	281	1.90	1.02	−0.57	1.36
Ideal state	293	2.88	1.08	2.17	3.79
*5. Occupation*					
High-level needs	52	0.17	1.09	NONE	−1.37
Some needs	98	0.73	0.51	−1.14	0.28
No needs	227	2.16	0.84	−0.38	1.83
Ideal state	287	3.11	0.93	1.52	3.84
*6. Control over daily life*					
High-level or some needs^a^	183	0.89	0.88	NONE	−0.02
No needs	311	2.35	0.96	−1.46	2.58
Ideal state	167	3.33	0.99	1.46	5.18
*7. Personal safety*					
High-level or some needs^a^	6	−0.91	0.79	NONE	−3.71
No needs	65	0.70	1.21	−0.95	−1.57
Ideal state	606	2.25	0.94	0.95	0.58
*8. Dignity*					
High-level or some needs^a^	22	0.47	1.57	NONE	−2.63
No needs	194	1.50	1.20	−1.42	−0.07
Ideal state	443	2.51	1.20	1.42	2.49

^a^Collapsed categories

**Table 7:** Rating scale diagnostics for ASCOT-Proxy-Resident

	Observed count	Observed average	OUTFIT MNSQ	Rasch-Andrich threshold	Category measure
*1. Food and drink*					
High-level needs	< 5*	−0.21	2.71	NONE	−4.00
Some needs	< 36*	0.03	1.03	−2.12	−1.78
No needs	126	1.49	1.47	0.06	0.39
Ideal state	300	2.53	1.07	2.06	2.54
*2. Home comfort & clean*					
High-level or some needs^a^	9	−1.08	0.68	NONE	−3.61
No needs	142	0.98	0.73	−1.89	−0.60
Ideal state	311	2.65	0.85	1.89	2.41
*3. Personal comfort & clean*					
High-level needs	< 10*	−2.67	0.28	NONE	−4.46
Some needs	< 10*	−0.54	0.88	−1.57	−2.73
No needs	108	0.98	0.83	−0.74	−0.68
Ideal state	344	2.45	1.00	2.31	1.90
*4. Social participation*					
High-level needs	15	−0.40	1.00	NONE	−2.63
Some needs	67	0.74	1.23	−1.83	−0.57
No needs	148	1.61	0.72	0.00	1.38
Ideal state	232	3.01	0.89	1.83	3.44
*5. Occupation*					
High-level needs	37	−0.07	0.83	NONE	−1.76
Some needs	123	1.08	0.74	−1.91	0.46
No needs	124	2.23	0.75	0.37	2.32
Ideal state	178	3.35	0.85	1.54	4.14
*6. Control over daily life*					
High-level needs	53	0.40	1.24	NONE	−1.18
Some needs	95	0.93	0.64	−1.38	0.62
No needs	142	2.32	1.00	−0.18	2.28
Ideal state	172	3.24	0.93	1.56	4.26
*7. Personal safety*					
High-level or some needs^a^	20	−0.38	1.08	NONE	−2.15
No needs	50	1.16	1.45	−0.20	−0.53
Ideal state	392	2.24	1.05	0.20	1.09
*8. Dignity*					
High-level	10	−0.04	1.72	NONE	−2.85
Some needs	36	0.59	1.09	−1.68	−1.12
No needs	206	1.78	1.45	−0.86	1.08
Ideal state	210	2.73	1.20	2.53	3.83

*Adjusted for report of small values (where observed count is < 5)

^a^Collapsed categories

Second, the average category measure and Rasch-Andrich category thresholds increased by response category for all ASCOT-SCT4 and ASCOT-Proxy-Resident items, so there was no evidence of disordered thresholds. However, the step difficulty increase by category did not fit into the criterion of between 1.4 and 5.0 logits for two ASCOT-SCT4 items (*Occupation* (0.76 logits) and *Social participation* (1.03 logits) at ‘some needs’ to ‘no needs’). For ASCOT-Proxy-Resident, there were inconclusive results for two items that did not meet the criterion (*Personal comfort and cleanliness* (0.83 logits) and *Personal safety* (0.40 logits)), but there were < 10 endorsements of high-level needs. This can cause instability of Rasch Andrich thresholds. Three ASCOT-Proxy-Resident items also did not meet the criteria with ≥ 10 endorsements (*Control over daily life* (1.20 logits) and *Dignity* (0.82 logits) at ‘some needs’ to ‘no needs’, *Occupation* (1.17 logits) at no needs to ideal state). Therefore, despite no evidence of disordered thresholds, there is evidence of less than optimal distinguishability at these thresholds.

Third, the OUTFIT MNSQ was within the criteria of < 2.0 for all ratings, except for ‘high level needs’ for ASCOT-Proxy-Resident *Food and drink*.

## Discussion

This analysis aimed to establish the structural validity of ASCOT-Proxy when completed by care home staff on behalf of older care home residents. Whilst ASCOT-SCT4 is thought to have a single factor structure based on previous studies (e.g., [15, 49]), this has not previously been assessed by care home staff report on behalf of older adults. On that basis, we also considered the structural validity of ASCOT-SCT4 collected in the ASCS by staff proxy report. We found that the single factor structure of the original ASCOT-SCT4 was replicated in this analysis. Therefore, we also expected to observe a single factor structure in the two measures of the ASCOT-Proxy collected in the DACHA study, an adapted version of the SCT4 for proxy-report. However, we found that only the ASCOT-Proxy-Resident had a single factor structure using EFA, whereas the ASCOT-Proxy-Proxy had a two-factor solution. The finding that the ASCOT-Proxy only forms a single factor solution for ASCOT-Proxy-Resident, not ASCOT-Proxy-Proxy, aligns to the first validation study of ASCOT-Proxy, conducted with data collected from family carers of community-dwelling adults with dementia [25]. The main difference between this previous study and the analysis presented here is that *Dignity* loads onto the higher-order factor for ASCOT-Proxy-Proxy and onto the single factor for ASCOT-Proxy-Resident with a loading > 0.4; it did not in the previous study [25]. This finding supports the retention of the *Dignity* item in the ASCOT-Proxy, which is part of the ASCOT-SCT4 (from which ASCOT-Proxy was developed) and was found to be conceptually important in its early development and testing [15].

In addition, aligning with the previous study [25], we recommend that, although both ASCOT-Proxy perspectives may be used to collect data, especially since qualitative evidence indicates that the dual proxy-proxy and proxy-resident perspective ratings enhance the measure’s acceptability [13], only the ASCOT-Proxy-Resident ought to be used in analysis of residents’ SCRQoL. This is because the ASCOT-Proxy-Resident maintains the structure of the original ASCOT-SCT4, with a single factor related to social care-related QoL (see [15] and analysis in this paper), whereas the eight ASCOT-Proxy-Proxy items form two separate measures. In addition, conceptually, the ASCOT-Proxy-Resident aligns more closely with the intended construct and purpose of ASCOT, as a measure of SCRQoL from a person-centred perspective and based on Sen’s capability approach [15, 50], since it invites a person-centred perspective in QoL rating by the proxy respondent. Using the ASCOT-Proxy is also preferable to the SCT4, without adaptation, for proxy-report, since it gives clearer indication that it is designed and intended for proxy-report, and has been found to be more acceptable and feasible for completion by both family carer and care staff proxy respondents [13, 23, 24].

The Rasch analysis indicated that there was acceptable model fit for ASCOT-Proxy-Resident with good internal consistency, overall model fit and item fit (as also, for ASCOT-SCT4 as a comparator). However, there was evidence of less than optimal distinguishability at the thresholds between outcome states, especially between some and high-level needs, for multiple items in both—SCT4 and—Proxy-Resident measures. There was also evidence of disordered thresholds, which appeared to be related to low frequency of selecting high-level needs. In applying psychometric approaches, it would usually be recommended to review these items to adjust item wording or response states to make it easier to choose the lowest QoL option or add new items. However, this study is a validation of an adapted version (ASCOT-Proxy) of an established measure (ASCOT-SCT4), with analysis of data collected in the same context (i.e., older adult care homes, by staff proxy) as a comparator. Therefore, rather than suggest further adaptations to either measure, we note these issues, especially the low frequency of selecting high-level needs, and set out our rationale for doing so, below.

ASCOT-SCT4 measure was designed as a preference-based measure for economic evaluation of social care interventions, service delivery and policy, although it is also used in other ways, e.g., assessment and care planning [51, 52]. The four-level response states are important as they enable the rating of QoL now and also, ‘what could be’ if services were no longer available (counterfactual). This allows the application of a counterfactual self-estimation method using an interview version, ASCOT-INT4, to estimate the impact of social care on quality of life [53]. The English context is covered by legislation, the Care Act (2014), which places a legal duty on local authorities to respond to adults’ eligible care needs. This welfare safety net means that it is uncommon for high-level needs to remain unmet. It is important, however, for the option of high-level needs to remain in the ASCOT measures to be able to identify the counterfactual (i.e., what it would be like if the system were no longer there and needs went unmet). The full range of ratings (ideal state to high-level needs) is also intended to allow measurement of trends, over time or by region, area or service, in long-term care systems that indicate stress-related failure or reduced performance. Therefore, we propose to keep the four response levels, despite the issue with low frequency of selection of high-level needs in this study, since it is part of the conceptual basis and design of ASCOT, and has also been observed with ASCOT SCRQoL collected by self-report (SCT4) or mixed-methods (CH4) [15, 16, 20], which indicates it is unlikely to be (at least, primarily) due to proxy report bias and is part of the intended concept/construct of ASCOT.

The study had a number of limitations. First, due to the high % missing data for demographics (e.g., sex—see Table 1) in DACHA, since the study drew on data inputted by care home staff as part of routine care, we were not able to consider differential item functioning. This ought to be considered in future studies, with more complete demographic data. Second, the secondary data from the ASCS survey, only had very limited data on proxy report (i.e., whether the proxy was care staff or someone else—family or friend). The DACHA study applied consistent guidelines that measures ought to be completed by direct care staff, who knew the person well, but detailed demographic or role-related data was not collected. Therefore, future studies may usefully consider the impact (if any) of these characteristics on the measures’ psychometric properties. This is important as previous studies have found some effect of the type of proxy (staff, family) and also characteristics of the proxy, especially how well they know the person [24]. Finally, it would have been interesting to directly compare ASCOT-Proxy and ASCOT-SCT4 (by proxy and self-report, where possible, although it is likely that many residents will not be able to self-report [20]) collected from the same care home residents, with the same proxy respondents. This was not possible in this study, due to the limitations of the data collection, which is why we applied the ASCOT-SCT4 collected from a comparable population (care home residents) and by the same approach (care home staff proxy).

Despite these limitations, the analysis provides evidence of the structural validity of ASCOT-Proxy-Resident completed by care home staff and indicates directions for future research. Since this analysis only reports one aspect of ASCOT-Proxy’s psychometrics (i.e., structural validity), further investigation is needed, and is planned with the DACHA study, to consider other measurement properties, e.g., internal reliability, construct validity by hypothesis testing. ASCOT-Proxy-Proxy does not retain the single factor structure of the original ASCOT-SCT4, which replicates a recent study of proxy report by family carers [25]. On the basis of these two studies, we do not recommend ASCOT-Proxy-Proxy’s use as a proxy-report measure of ASCOT SCRQoL, as it does not form a unidimensional scale of SCRQoL. However, we recommend that ASCOT-Proxy-Proxy data still be collected alongside ASCOT-Proxy-Resident, using the current ASCOT-Proxy questionnaire format (see Fig. 1), to enhance feasibility and acceptability of ASCOT-Proxy data collection and to give insight into whether, and how, ratings differ between proxy perspectives by item [13]. Qualitative evidence of the feasibility and acceptability of ASCOT-Proxy data collection, specifically with care home staff, would give further insight into the best approach to data collection or further adaptation of the questionnaire for this context.

### Supplementary Information

Below is the link to the electronic supplementary material.

Supplementary file1 (JPG 153 KB)

Supplementary file2 (JPG 151 KB)

Supplementary file3 (DOCX 76 KB)

## Data Availability

The data are not publicly available due to data protection and ethical considerations. Please contact the corresponding author to discuss reasonable requests for access to the data that support the findings of this study.

## Footnotes

1 By structural validity, we refer to the degree to which the scores of a scale are an adequate reflection of the dimensionality of the construct to be measured [14].

2 Per ASCS year (excluding 2021): Mean 62.8, Std. Dev. 11.68, and Range 40 to 80. The 2021 ASCS was voluntary, rather than mandatory. There were only six proxy responses by care home staff.
