# Supplementary figures and images for: Exploratory factor analysis and Rasch analysis to assess the structural validity of the Adult Social Care Outcomes Toolkit Proxy version (ASCOT-Proxy) completed by care home staff
Source: Qual Life Res. 2024 Mar 20;33(6):1555–67. doi: 10.1007/s11136-024-03631-1 (PMC11116179; doi:10.1007/s11136-024-03631-1)

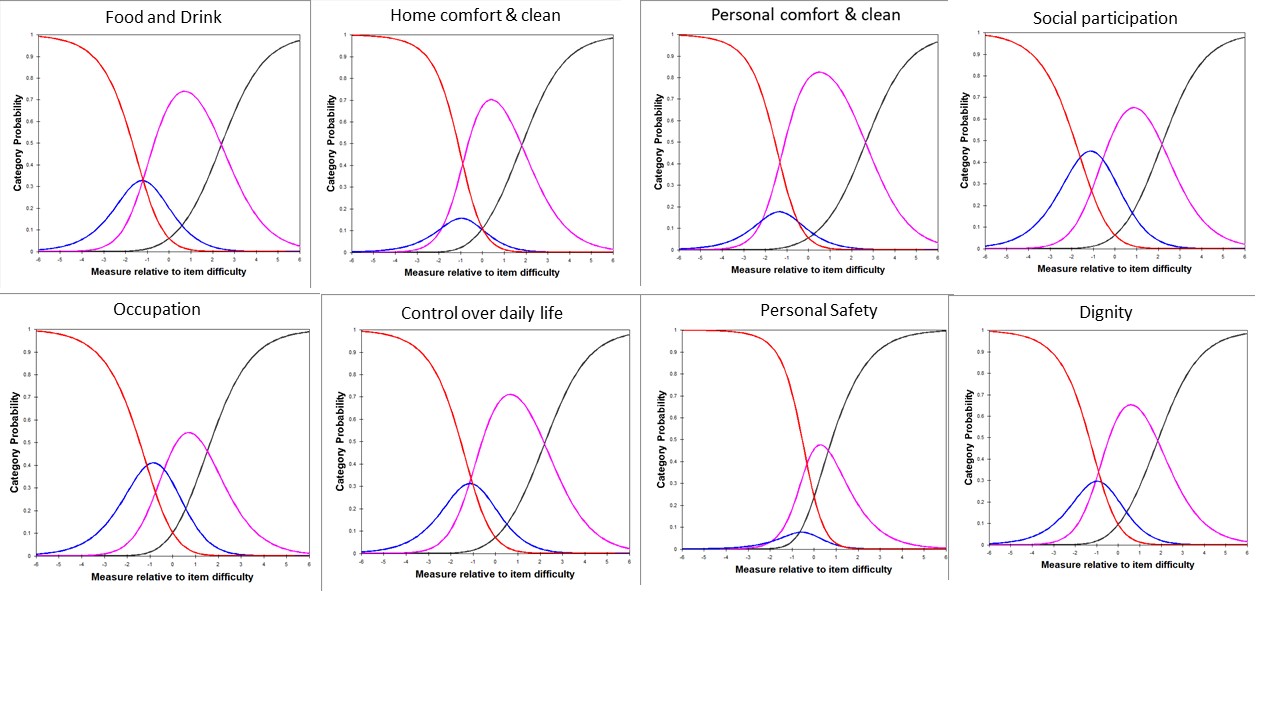

Supplement: Supplementary file 1 — Supplementary file1 (JPG 153 KB) [file 11136_2024_3631_MOESM1_ESM.jpg]

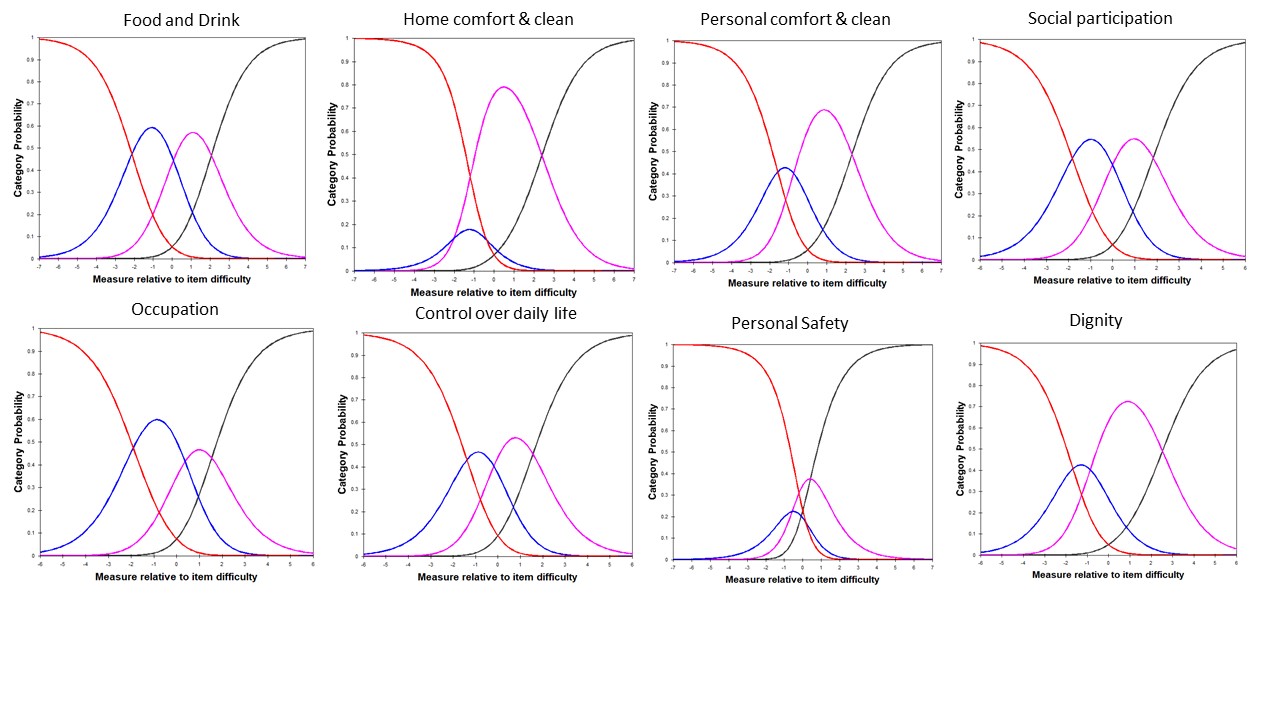

Supplement: Supplementary file 2 — Supplementary file2 (JPG 151 KB) [file 11136_2024_3631_MOESM2_ESM.jpg]
