# Supplementary material for: Exploratory factor analysis and Rasch analysis to assess the structural validity of the Adult Social Care Outcomes Toolkit Proxy version (ASCOT-Proxy) completed by care home staff
Source: Qual Life Res. 2024 Mar 20;33(6):1555–67. doi: 10.1007/s11136-024-03631-1 (PMC11116179; doi:10.1007/s11136-024-03631-1)
Supplement: Supplementary file 3 — Supplementary file3 (DOCX 76 KB) [file 11136_2024_3631_MOESM3_ESM.docx]

**Supplemental Item: Winsteps Table 23.99 – standardized residual correlations**

**Study 1 (ASCS). ASCOT-SCT4**

*
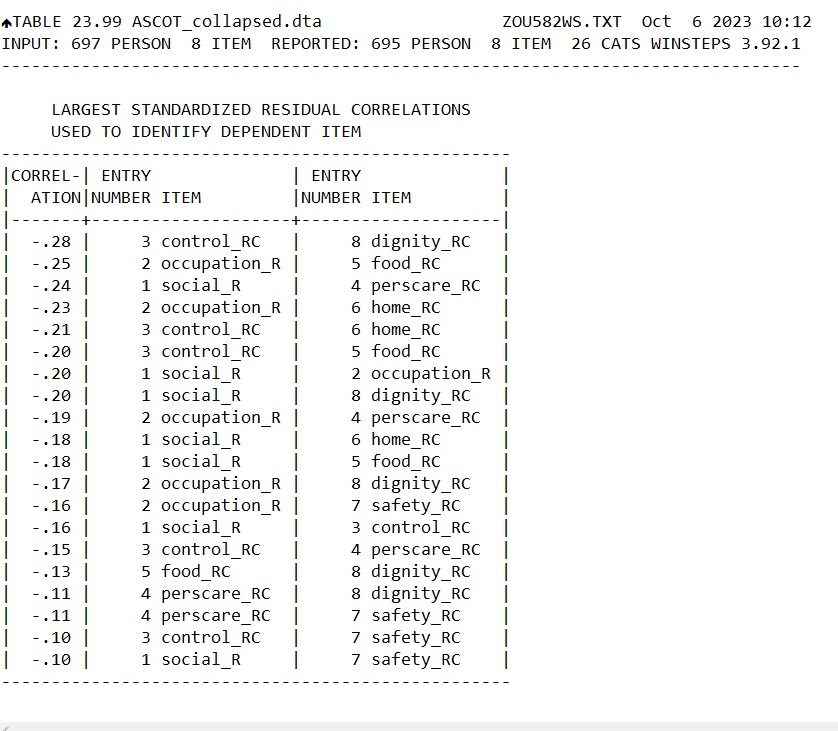
*

**Key to Item labels:**

1 Social_R ASCOT-SCT4 Social participation

2 Occupation_R ASCOT-SCT4 Occupation

3 Control_RC ASCOT-SCT4 Control over daily life (collapsed categories)

4 perscare_RC ASCOT-SCT4 Personal comfort and cleanliness (collapsed categories)

5 food_RC ASCOT-SCT4 Food and drink (collapsed categories)

6 home_RC ASCOT-SCT4 Accommodation comfort and cleanliness (collapsed categories)

7 safety_RC ASCOT-SCT4 Personal safety (collapsed categories)

8 dignity_RC ASCOT-SCT4 Dignity (collapsed categories)

**Study 2 (DACHA). ASCOT-Proxy-Resident**

TABLE 23.99 ASCOT proxy resident.dta ZOU046WS.TXT Jan 11 2024 10:47

INPUT: 462 PERSON 8 ITEM REPORTED: 462 PERSON 8 ITEM 30 CATS WINSTEPS 5.5.0.0

---------------------------------------------------------------------------------

LARGEST STANDARDIZED RESIDUAL CORRELATIONS

USED TO IDENTIFY DEPENDENT ITEM

-----------------------------------------------

|CORREL-| ENTRY | ENTRY |

| ATION|NUMBER ITEM |NUMBER ITEM |

|-------+------------------+------------------|

| .28 | 2 ascot_q2pR | 3 ascot_q3p |

|-------+------------------+------------------|

| -.34 | 1 ascot_q1p | 5 ascot_q5p |

| -.31 | 2 ascot_q2pR | 6 ascot_q6p |

| -.29 | 1 ascot_q1p | 6 ascot_q6p |

| -.26 | 2 ascot_q2pR | 5 ascot_q5p |

| -.26 | 4 ascot_q4p | 8 ascot_q8p |

| -.26 | 3 ascot_q3p | 6 ascot_q6p |

| -.21 | 3 ascot_q3p | 4 ascot_q4p |

| -.21 | 3 ascot_q3p | 5 ascot_q5p |

| -.21 | 5 ascot_q5p | 8 ascot_q8p |

| -.20 | 6 ascot_q6p | 8 ascot_q8p |

| -.20 | 4 ascot_q4p | 6 ascot_q6p |

| -.18 | 1 ascot_q1p | 4 ascot_q4p |

| -.17 | 5 ascot_q5p | 7 ascot_q7pR |

| -.16 | 2 ascot_q2pR | 8 ascot_q8p |

| -.13 | 1 ascot_q1p | 8 ascot_q8p |

| -.13 | 4 ascot_q4p | 7 ascot_q7pR |

| -.12 | 3 ascot_q3p | 7 ascot_q7pR |

| -.11 | 7 ascot_q7pR | 8 ascot_q8p |

| -.09 | 6 ascot_q6p | 7 ascot_q7pR |

-----------------------------------------------

**Key to Item labels:**

1 ascot_q1p ASCOT-Proxy-Resident Food and drink

2 ascot_q2pR ASCOT-Proxy-Resident Accommodation comfort and cleanliness (collapsed categories)

3 ascot_q3p ASCOT-Proxy-Resident Personal comfort and cleanliness

4 ascot_q4p ASCOT-Proxy-Resident Social participation

5 ascot_q5p ASCOT-Proxy-Resident Occupation

6 ascot_q6p ASCOT-Proxy-Resident Control over daily life

7 ascot_q7pR ASCOT-Proxy-Resident Personal safety (collapsed categories)

8 ascot_q8p ASCOT-Proxy-Resident Dignity
